# Hyaluronan–Itaconic Acid–Glutaraldehyde Films for Biomedical Applications: Preliminary Studies

**DOI:** 10.3797/scipharm.1504-17

**Published:** 2015-07-29

**Authors:** Javier Adrián Calles, Jorge Aníbal Ressia, Juan Manuel Llabot, Enrique Marcelo Vallés, Santiago Daniel Palma

**Affiliations:** 1 PLAPIQUI-CONICET, Universidad Nacional del Sur, Camino La Carrindanga Km 7, 8000, Bahía Blanca, Argentina; 2 Dpto Biología Bioquímica y Farmacia, Universidad Nacional del Sur, San Juan 670, 8000, Bahía Blanca, Argentina; 3 Institute of Applied Ophthalmobiology, University of Valladolid, Paseo de Belén 17, 47011, Valladolid, Spain; 4 Comisión de Investigaciones Científicas de la Provincia de Buenos Aires (CIC), 1900, La Plata, Argentina; 5 UNITEFA-CONICET, Universidad Nacional de Córdoba, Haya de la Torre y Medina Allende, X5000HUA, Córdoba, Argentina

**Keywords:** Hyaluronan, Cross-link, Polymers, Biomaterials

## Abstract

New hyaluronic acid–itaconic acid films were synthesized as potential materials with biomedical applications. In this work, we explored the homogeneous cross-linking reactions of hyaluronic acid using glutaraldehyde in the presence of itaconic acid and triacetin as plasticizers.

Biomechanical properties were assessed in terms of stability by measuring swelling in aqueous environments, investigating wettability using contact angle tests, and evaluating bioadhesive performance. The ductility of the materials was evaluated through stress-strain measurements and the morphology was explored by scanning electron microscopy.

The results show that the incorporation of itaconic acid improved most of the desirable properties, increasing adhesiveness and reducing wettability and swelling. The use of triacetin enhanced the strength, bioadhesiveness, and ductility of the material.

## Introduction

The hyaluronic acid (HA), also known as hyaluronan, is a naturally occurring high molecular mass linear biopolymer, consisting of alternating units of *N*-acetyl-β-D-glucosamine and β-D-glucuronic acid. It is a biodegradable, biocompatible, non-toxic, non-immunogenic and non-inflammatory biomaterial [1]. The total amount of HA in the adult human has been estimated to be 11–17 g [2]. It is distributed throughout the human body, forming part of the synovial fluid and the extracellular matrix of biological tissues such as the skin, umbilical cord and vitreous humor. In medical practice, HA is widely used in many pathological conditions such as osteoarthritis [3], wound repair [4] and eye surgery [5].

HA has excellent water-binding capacity, responsible for retaining moisture in the eyes, joints and skin [6]. Dissolution of this biopolymer results in a highly viscous gel with unique viscoelastic properties, which enable its use in orthopedics [7]. Many studies have been performed to formulate HA in an injectable form [8], which can be used to treat osteoarthritis of the knee [9]. Several HA derivatives have been developed for drug delivery [10], mainly for its potential as a biodegradable carrier [11]. Some authors have reported the use of this polymer for different proteins, drugs [12], peptides [13] or for gene delivery [14, 15] using HA as a depot system [16], as hydrogels (physically and chemically cross-linked) [17–19] or as nano- or micro-particulate systems [20, 21]. Studies related to biocompatibility and biodegradability [22] have supported the use of HA as a promising biomaterial to design modified drug delivery systems.

The poor stability of HA in living tissues and fast dissolution in water make some modifications necessary to overcome these drawbacks. Cross-linking reactions have been carried out in order to improve HA properties, leading to more robust materials. Heterogeneous cross-linking methods have been reported, where reactions were carried out on previously casted solid HA films or membranes, using glutaraldehyde (GTA) as the cross-linker [23]. However, this processes not only improves the strength of the cross-linked materials, it also makes them more rigid and fragile (brittle). Further techniques as well as polymer interpenetration have also been reported [24, 25].

In this work, we explored chemical modifications to the HA backbone by a homogeneous cross-linking reaction between HA and GTA in the presence of itaconic acid (IT), a naturally occurring compound that is non-toxic, and readily biodegradable. The homogeneous reaction was achieved by adding the cross-linker agents to the HA solution, obtaining a final cross-linked film after casting.

To mitigate the fragility of cross-linked films, triacetin (TA) was assessed as a potential plasticizer in order to obtain more ductile materials. The stability of the materials in aqueous medium was evaluated through swelling and water affinity was studied using contact angle assays. The ductility and bioadhesive force were appraised through stress-strain tests. In addition, the morphology was studied by scanning electron microscopy (SEM).

## Experimental

### Materials

Hyaluronic acid sodium salt (Mw: 1,560,000 Da) was obtained from Parafarm® (Buenos Aires, Argentina), itaconic acid for synthesis was provided by Merck Schuchardt OHG (Hohenbrunn, Germany), and glutaraldehyde (25% in H_2_O) and porcine mucin were purchased from Sigma-Aldrich Corp. (St. Louis, MO, USA). All other chemicals were of extra pure reagent grade and used as received.

### Film Synthesis

Four HA systems (Table 1) were synthesized from HA/GTA, HA/IT/GTA and HA/GTA + TA solutions, using bi-distilled water as the solvent. The amount of each reactant was set to achieve (1:2) and (1:4) HA/GTA molar ratios, or (1:1:2) in the case of the HA/IT/GTA solutions. All HA concentrations were adjusted to 1% (w/w). The plasticizer, TA, was used in order to attain 20% of the solid weight of the final film.

**Tab. 1:**
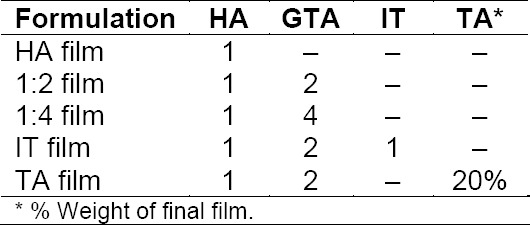
Composition of films (molar ratios)

Cross-linking reactions were accomplished under acidic conditions; the pH value was adjusted to 3.7 with 0.01 M HCl. After 24 h of reaction time under slight stirring at room temperature (RT), the obtained gels were cast at RT under an extractor hood. Different sized Petri dishes were used to obtain a variety of film thicknesses.

### Contact Angle Measurements

The wetting behavior of film surfaces was characterized by the contact angle (CA) method. Measurements were conducted with an Easy Drop DSA goniometer from Kruss GmbH (Hamburg, Germany), using a distilled water drop at RT.

An imaging system was used to measure the CA; the shape and size of water droplets on the tested surfaces of films was measured by digital image analysis software. The CA from the images was measured 5 sec after a 5 μL droplet of distilled water was placed on the sample surface. Three samples (n=3) from each film were used for CA measurements.

### Film Swelling

The determination of swelling behavior of the films was carried out by adapting the method described by Llabot et al. [26]. Cross-linked films were tested in distilled water at RT after sample drying to achieve a constant weight (n = 3 in triplicate). The swelling ratio (SR) was calculated using the equation: SR = Ws/Wd, where Ws is the weight of the sample at equilibrium at each temperature and Wd is the weight of the dried sample.

### Mucoadhesion and Stress-Strain

The adhesive bond was measured in terms of the strength needed to separate or break the adhesive bonds between the matrix and the biological substrate. Samples were attached to a holder with cyanoacrylate-based glue, and the support was suspended from a spring placing the free surface of the film in direct contact with the biological substrate, after pre-wetting of the area with 50 μl of water. A force of 20 grams was applied for 30 seconds before performing the measurement on an adapted Jolly balance. As the biological substrate, a 30% (w/v) porcine gastric mucin gel was used.

The stress-strain properties of the films were studied using sample strips of 1 × 5 cm (n = 10) on an Instron 3369 tester (Norwood, USA) in traction mode at 2 mm/min at RT (23°C).

### Morphological Characterization

The surface morphology of samples was examined by SEM (Leo EVO-40XVP from Carl Zeiss AG, Oberkochen, Germany). The samples were swelled in distilled water for a certain period of time at room conditions. Subsequently, they were freeze dried and coated with Au in a PELCO 91000 sputter coater before observation.

### Statistical Analysis

The results of the experiments are expressed as mean ± standard error. Differences were considered to be significant when p ≤ 0.05. Significant differences were determined by Student’s *t*-test.

## Results and Discussion

### Contact Angle and Swelling

**Fig. 1:**
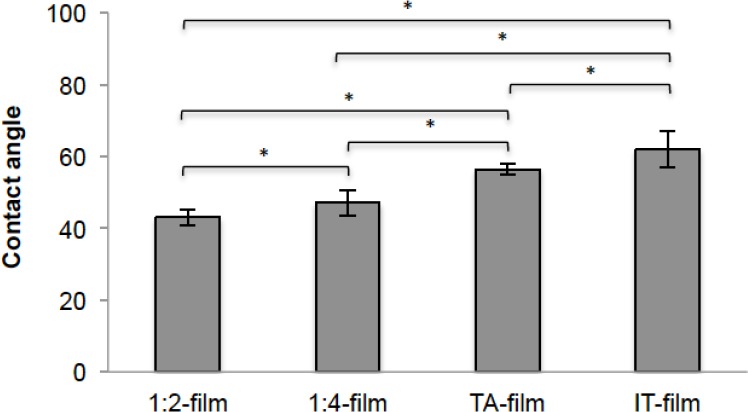
CA values for different cross-linked HA films

CA values are presented in Figure 1. Significant differences (p < 0.05) were found between all groups. The data show an increase in CA when the GTA concentration was higher in the film (1:2 film and 1:4 film). Similarly findings were observed when TA (hydrophobic plasticizer) and IT were added; the presence of TA led to a greater CA, evidencing an increase in this parameter which shows that these compounds directly affect the interaction between the system and the surrounding aqueous environment.

**Fig. 2:**
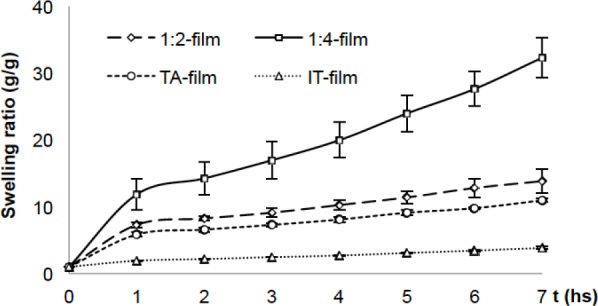
Swelling ratio of cross-linked HA films immersed in distilled water at RT

Swelling measurements (Figure 2) are useful to evaluate the stability and cross-linking rate of films. Although, as shown in Figure 1, the use of a higher cross-linker concentration led to materials with reduced wettability, the swelling studies showed poor stability for these formulations in aqueous media (considerable swelling). On the other hand, the presence of TA in the film led to similar behavior with respect to the same formulation without the plasticizer (1:2 film). The films containing IT in the formulation had the lowest swelling ratio and the highest CA measurements, suggesting that IT increases the cross-linking ratio between HA chains. Moreover, the trend showed by the IT films regarding swelling behavior suggests lower water contents than those reported by Tomihata et al., i.e. 60% after 24 h of swelling in PBS for heterogeneous HA cross-linked films [23].

### Mucoadhesion, Stress-Strain and Morphological Characterization

The adhesive strength of each formulation is shown in Table 2. An evident increase in mucoadhesion was observed when HA was cross-linked with GTA. The addition of both IT and TA also improved the adhesion strength.

**Tab. 2:**
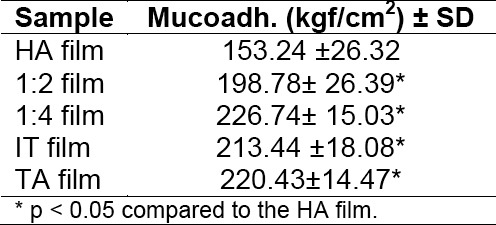
Mucoadhesive strength

The higher increase obtained with TA was attributed to the rise in chain mobility favored by the presence of the plasticizer, which contributed to the interaction between HA and mucin chains. In general, the introduction of a cross-linker results in a higher Tg, which in turn decreases bioadhesion through a decrease in chain mobility and interpenetration [27]. However, in our case, the bioadhesive force of the HA cross-linked films was increased. This can be attributed to the fact that increased cross-linking density results in less swelling, leading to a greater number of carboxyl groups in a given surface area for bioadhesion. Thus, the formation of secondary hydrogen bonds is facilitated [28].

**Fig. 3:**
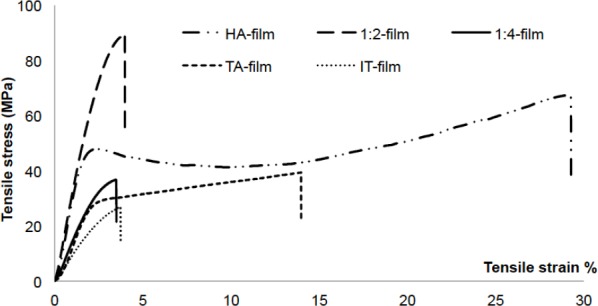
Stress-strain assay of cross-linked films at RT

Stress-strain studies (Figure 3) revealed a marked change in the tensile behavior of the cross-linked films, changing from ductile to brittle; in fact, these films broke before reaching the yield point. In contrast, cross-linked films containing TA exhibited plastic deformation. Although the cross-linking process led to increased polymer rigidity as a result of the restrained movements of the polymer molecules, the presence of TA mitigated these effects. Hence, the presence of TA gives the possibility of cross-linking HA without losing flexibility.

**Fig. 4:**
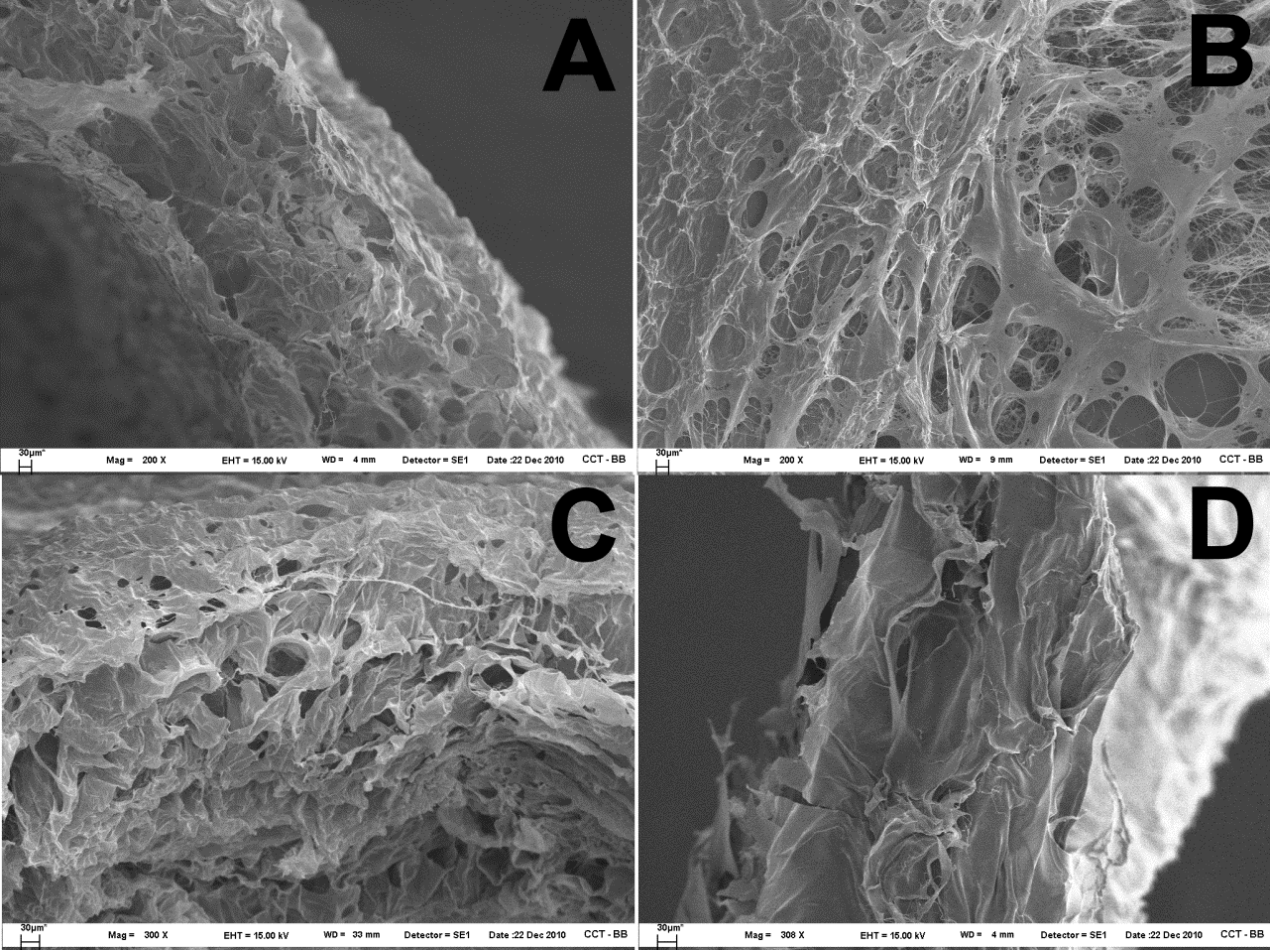
SEM pictures of swelled a) 1:2 film (200x), b) 1:4 film (200x), c) IT film (300x) and d) TA film (300x)

The SEM images (Figure 4) show the surface structure of the swelled films. All formulated cross-linked films showed a porous structure after swelling in aqueous media. This property could be of interest for scaffolds and tissue engineering purposes.

The importance of new biomaterials for medical applications is observed in a consistently growing research literature [29–34]. Most current research efforts are focused in the development of biomaterials from naturally occurring polymers [35–39]. However, many of these materials have poor biomechanical properties and insufficient stability in aqueous environments and living tissues. Thus, the need for more robust and stable materials has led to the study of different structural modifications on its backbone.

The results obtained in this study clearly show that cross-linking HA affects system interactions with the surrounding aqueous medium. The contact angle and swelling measurements shown in Figures 1 and 2 demonstrate that cross-linking of HA chains provided insoluble matrices capable of taking up water inside its structure without losing stability. Similar findings have been reported for HA [10, 23, 40] and other natural biopolymers like chitosan [41, 42], alginate [43, 44] and collagen [45].

Some desirable properties of natural biomaterials like biocompatibility, biodegradability, ductility and bioadhesiveness can be altered after chemical structural modifications. Ductile behavior not only guarantees better handling of the material but also makes it more suitable and adaptable to living tissues; this is complemented by the ability to remain attached to the mucosal layers of tissues. The results of this work confirm that cross-linking chemical reactions increase the sturdiness of biopolymers like HA but often lead to more fragile materials. However, the use of appropriate plasticizers can overcome these drawbacks [46, 47] and also improve other properties like bioadhesion.

Previous reports have noted the toxicity of GTA [48], which can be avoided by extensive washing [10] or inactivation [49].

## Conclusion

The use of GTA as a cross-linking agent significantly improved the integrity and the bioadhesiveness of HA films in aqueous environments. Cross-linked polymers showed more stability and increased porosity when the material had swelled.

Incorporation of IT improved most of the desirable properties studied for the HA/GTA systems, i.e. it increased adhesiveness and reduced wettability and swelling, but also made the original material more fragile. On the other hand, the use of TA as a plasticizer not only enhanced the strength and the bioadhesiveness of the material, but also made it more ductile and less rigid. The use of GTA as a cross-linker was found to be effective at improving HA properties; however, more studies should be performed regarding toxicity.
